# Naturalist's Monthly Report

**Published:** 1811-07

**Authors:** 


### NATURALIST'S MONTHLY REPORT.

### MAY.

#### LEAFING MONTH.

Hail! bounteous May, that dost inspire
Mirth and youth and warm desire;
Woods and groves are of thy dressing,
Hill and dale doth boast thy blessing.

On the 1st of the month the wind was south-west; from the 2d to the 4th westerly; from the 5th to the 11th south-west; on the 12th and 13th south-east; on the 14th south and south-west; on the 15th and 16th south-east; on the 17th and 18th easterly; from the 19th to the 23d easterly; on the 23d and 24th south-west; from the 25th to the 27th southerly; on the 28th and 29th west; on the 30th south; and on the 31st south-east.

We had a heavy gale of wind, accompanied with showers, on the 5th, and strong gales on the 2d, 6th, 14th, 19th, 28th, and 29th.

The only thunder storm in the course of the month was in the morning of the 12th, and it was of short duration.

We had rain on the 1st, 3d, 5th, 6th, 7th, 8th, 9th, 10th, 11th, 15th, 16th, 24th, 28th, and 31st. The weather has not been so hot as it frequently is in the month of May.

May 2d. Toads begin to croak in the evenings.

The Swifts are now seen in considerable numbers, and fly screaming after each other in the same manner as they do in the middle of summer.

May

May 4th. The nightingale is arrived.

Sweet-scented vernal grass *(anthoxanthum odoratum)* is in flower. It is this grass, chiefly, that gives to hay its peculiar scent.

May 6th. The Black-cap sings.

The Cuckoo is arrived. Mushrooms are gathered.

May 7th. Chaffers *(scarabæus melolontha)* begin to fly about in the evenings. It is really wonderful to observe with what exactness of time the first leafing of the trees, and the emerging of these insects from the ground, take place. Whether the season be early or late, the Chaffers invariably make their first appearance as soon as a sufficiency of food is provided for them by the vernal foliage.

May 8th. The seven-spotted lady-bug *(coccinella septempunctata)* flies about. The bloom of the hawthorn begins to expand.

Damson trees are in bloom.

Yellow wagtails *(motacilla flava)* appear.

May 12th. This was a close, damp, and yet sultry day. The ponds and manure heaps along the sides of the road were extremely offensive.

The caterpillars of the barred tree, lackey moth *(bombyx neustrius* of Haworth) begin to emerge from the ova which the parent insects deposited in the autumn round slender twigs of apple trees. These caterpillars are in some seasons so numerous as to devour a great part of the foliage.

There has of late been so much rain in the country to the westward, that the rivers have overflowed their banks.

May 13th. The sowing of barley, which was much retarded by the wet weather, is now going on; and, if the weather continues fine for a few days longer, will be finished.

May 15th. Bird's--foot trefoil *(ornithopus perpusillus)*, heart medick *(medicago polymorpha)*, common vetch *(vicia sativa)*, and common bird's-foot trefoil *(lotus corniculatus)*, are in flower. The hawthorn also is now in full bloom.

May 20th. The Chaffers are not at all numerous; but, if we may judge by the devastations which have of late been committed by the chaffer grub (or rook-worm, as it is usually called) it seems probable that in the next spring these insects will be unusually abundant.

The froth-worm, or cuckoo-spit, appears on the blades of grass and other herbage.

The leaves of the mulberry tree are not yet fully expanded. Those of the walnut tree have been much injured by the chaffers.

May 26th. Wall butterfly *(papilio mægera)*, red admiral *(papilio atalanta)*, and fern chaffer *(scarabæus horticola)*, appear.

May 27th. Young wood pigeons are nearly fledged.

Rye is in full ear; and the bramble, and dog-rose, are in flower.

May 30th. Green peas, and ripe strawberries gathered.

May 31st. The crops of grass are heavier in this neighbourhood than they have been for several years past. The rye also promises to afford an abundant crop; and the wheat and barley are, on the whole, looking very well.

The yellow iris, and fox glove are in flower.

*Hampshire.*

